# Cognitive and Socioenvironmental Factors Associated with Suicide Ideation in Adolescents

**DOI:** 10.1007/s10964-025-02307-4

**Published:** 2026-01-05

**Authors:** Amy Brausch, Taylor Kalgren, Andrew Littlefield, Chelsea Howd, Madeline Wildman

**Affiliations:** 1 Department of Psychological Sciences, Western Kentucky University, 1906 College Heights Blvd, Bowling Green, KY 42101 USA; 2 Department of Psychological Sciences, Texas Tech University, Lubbock, TX, 79409 USA

**Keywords:** Adolescent suicide, Suicidal ideation, Adolescents, Risk factors, Environmental factors

## Abstract

Existing research on adolescent suicide risk has focused on homogenous cognitive and psychiatric risk factors and not as much on socioenvironmental factors. Data are also lacking for adolescents in rural communities who may experience different risk and protective factors than youth in urban areas. The current study examined multiple cognitive and socioenvironmental factors and their association with current suicide ideation severity in a sample of adolescents. The sample included 458 adolescents (50.8% female; ages 14–18; mean age = 15.64) recruited from public high schools in rural and under-resourced communities. Self-report measures were administered assessing suicide ideation, cognitive factors (defeat, entrapment, grit, self-efficacy), and socioenvironmental factors (SES, social support, food insecurity, lapses in medical care, and firearm access). All factors were significantly correlated with suicide ideation in expected directions using bi-variate correlations, except for access to firearms. However, in multivariate analyses, defeat and entrapment showed the strongest associations with suicide ideation, and food insecurity was the only other significant variable. Overall, defeat and entrapment demonstrated the strongest positive association with current suicide ideation severity in bi-variate and multi-variate analyses. These findings underscore how defeat, entrapment, and food insecurity are salient risk factors for suicide ideation in adolescents from rural areas and seem to be more impactful than other cognitive and environmental factors.

## Introduction

It is crucial to address suicide thoughts and behaviors in adolescents because suicide is the second leading cause of death in youth and is the only cause increasing within the past 50 years (CDC, 2025a) despite growing public-health awareness. Rates of suicide ideation and attempts increase from childhood to adolescence, and the rates for death by suicide increase considerably from the 10–14 age range to the 15–19 age range (2.3 to 9.8 per 100,000, respectively; Drapeau & McIntosh, 2025). These increases signal that the transition into adolescence may be a prime developmental stage for the emergence of self-harm thoughts and behaviors. Results from the most recent Youth Risk Behavior Surveillance System in the United States found that 9% of high school students reported attempting suicide in the past year. Suicide ideation also remains a significant concern as almost 20% of high school students reported seriously thinking about suicide in the past year (CDC, 2025b). More than one-third of adolescents who report thoughts of suicide transition to a suicide plan and/or attempt, with 60–70% of these transitions occurring within one year of suicide ideation onset (Glenn & Nock, 2014; Nock et al., 2013). Decades of research on suicide risk factors have focused on depression, hopelessness, and psychiatric diagnoses. It is acknowledged that studies of traditional risk factors are redundant and not effective at predicting suicide (Fox et al., 2015; Franklin et al., 2017), and most studies tend to focus on cognitive and psychological factors rather than socioenvironmental factors (Gallagher & Miller, 2018). The current study examines multiple cognitive (defeat, entrapment, grit, and self-efficacy) and socioenvironmental (SES, firearm access, social support, and food security) factors and how they associate with current suicide ideation severity in a sample of adolescents recruited from rural, low-income, and health professional shortage communities.

One model of suicide risk that includes cognitive, emotional, and socioenvironmental factors is the Integrated Motivational-Volitional Model of Suicidal Behavior (IMV; O’Connor & Kirtley, 2018). The IMV model proposes a dynamic pathway through which risk for suicide ideation and attempts are conferred. The model includes both socioenvironmental factors that may increase one’s vulnerability to suicide ideation, and cognitive factors that may further increase risk of suicide ideation and suicide attempts. The model is centered on the cognitive and emotional experiences of defeat and entrapment, which are proposed to increase likelihood of suicide ideation and intent, and which may be strengthened or buffered by other factors like (lack of) coping, social support, and resilience. Access to lethal means is proposed to increase the likelihood of suicide ideation, intent, and attempts. A recent systematic review of articles evaluating the IMV model found that very few included samples of adolescents, and the majority tested the relationships between defeat, entrapment, and suicide ideation (Souza et al., 2024). Therefore, research on other components of the IMV model and suicide ideation is lacking, specifically for adolescents. The current study aims to further contribute to this literature by examining multiple cognitive and socioenvironmental factors as they relate to suicide ideation in adolescents.

### Cognitive Factors

Cognitive factors are internal to individuals and involve their perceptions, beliefs, and attitudes about themselves and their situation. The IMV model proposes several cognitive factors (beyond traditional factors like depression and hopelessness) that are significant to the experience of suicide ideation, namely defeat and entrapment, which are considered central to the framework. Defeat, defined as a psychological state stemming from helplessness associated with a failed struggle or loss of social status (Gilbert & Allan, 1998), has been found to significantly and directly associate with suicide ideation, and indirectly through entrapment (Souza et al., 2024). Entrapment can be experienced both internally and externally and is conceptualized as feeling trapped in psychological pain and/or trapped in a situation from which one feels powerless to escape (Gilbert & Allan, 1998). Defeat and entrapment have both direct and indirect associations with suicide thoughts and behaviors above and beyond other risk factors in various cross-sectional samples of adults (Siddaway et al., 2015; Silvestre Vidal et al., 2024). However, research examining defeat and entrapment in adolescents is limited. Pollak et al. (2021) examined these constructs as a single variable and found that defeat/entrapment was positively associated with suicide ideation severity in a community sample of adolescents. At 3-month follow-up, defeat/entrapment was not found to be a significant predictor of future suicidal ideation after controlling for depressive symptoms. In contrast, longitudinal research with adults has indicated that defeat, but not entrapment, predicted the frequency of suicidal ideation at 12-month follow-up, even after controlling for depressive symptoms (Taylor et al., 2011). These contrasting findings indicate the need for future research with adolescents. Two cross-sectional studies examined defeat in a community sample of Chinese adolescents and found it to be significantly and positively associated with suicide ideation (Ren et al., 2019; Yang et al., 2022). Entrapment was most strongly correlated with suicide ideation compared to anger, depression, resilience, and psychosomatic symptoms in sample of over 11,000 Korean adolescents (Park et al., 2010). Entrapment mediated the relationship between defeat and suicide ideation in a sample of Chinese adolescents, supporting its central role in the transition from defeat to suicidal thinking (Li et al., 2021). Additionally, perceptions of defeat and entrapment have been identified as mediators between both mental well-being and insomnia and suicidal ideation in Scottish samples, further highlighting their relevance as cognitive mechanisms contributing to risk for adolescents (Russell et al., 2018, 2020). These findings support the IMV model by reinforcing defeat and entrapment as critical cognitive risk factors for adolescent suicidal ideation in various international samples and emphasizing the need for additional research. The current study extends prior research by examining defeat and entrapment plus two additional cognitive constructs, self-efficacy and grit, which have emerging evidence as potential protective factors for suicidal ideation in a sample of American adolescents.

Self-efficacy, defined as the belief about one’s capability to accomplish tasks and succeed in life, is understudied in the context of suicide, but is a robust (negative) predictor of many health behaviors including substance use (Czyz et al., 2014; Hasking, 2017). The few studies examining self-efficacy in adolescents found that it associates with decreased suicide ideation (Valois et al., 2015; Wu & Yaacob, 2017), and that it is a buffer between both emotion reactivity and hopelessness and suicide ideation (Li & Kwok, 2023; Liu et al., 2021) in samples of Chinese adolescents aged 12–14 years. Self-efficacy is a promising, and potentially modifiable, protective factor for suicide ideation and behavior; clinical interventions that target self-efficacy in adolescents with suicide risk (Czyz et al., 2019) and school-based intervention programs that target overall resilience (Sabin et al., 2023) show meaningful gains. Adolescents with low self-efficacy may feel less capable of coping with difficult life circumstances, which can increase perceived stress (Piekarska, 2020) and intensify the impact of entrapment on suicidal thoughts. In contrast, those with high self-efficacy may perceive themselves as capable of addressing challenges (Urdan & Pajares, 2006), potentially reducing the likelihood that entrapment progresses into suicide ideation. Given its demonstrated protective effects and potential in targeted interventions, self-efficacy warrants further investigation as a possible factor in adolescent suicide prevention.

Grit, defined as passion and perseverance for working toward long-term goals (Duckworth et al., 2007), has also emerged as a potential protective factor for suicide ideation. Grit has been studied within the context of suicide risk, but thus far, most existing research has focused on adults. Grit negatively associates with suicide ideation and buffers the effects of hopelessness in samples of college students and military personnel (Clement et al., 2020; Pennings et al., 2015). Additionally, grit negatively associated with suicide ideation at baseline and a 4-week follow-up in college students (Kleiman et al., 2013). However, grit positively associated with more frequent suicide attempts in college students in a different study; suicide ideation was not assessed (Anestis & Selby, 2015). Findings on grit as a protective factor for suicide ideation in adults is consistent, but the association between grit and suicidal behavior may be different. Although most research among adult samples report grit as a protective factor, findings are mixed. It is also unclear the role that grit plays in adolescent samples given limited research. One study found grit to negatively associate with suicide ideation in a sample of Chinese adolescents who had at least one absent parent due to migrant work; grit also differentiated between those with and without suicide ideation (Liu et al., 2021). Grit has potential to be a modifiable factor that can be targeted in school-based programs to promote perseverance through difficult emotional and situational times (Sabin et al., 2023). Adolescents with high levels of grit may be less likely to develop suicidal thoughts when experiencing entrapment, as grit may promote a stronger future orientation and greater capacity to tolerate distress. Supporting this, grit has been associated with perceived goal progress and distress tolerance in adolescents (Schmahl & Nguyen, 2022) and weakened the relationship between negative life events and suicide ideation in adult samples (Blalock et al., 2015). As a modifiable protective factor, grit may be an important focus for early interventions and research aimed at reducing suicidal ideation in adolescents.

### Socioenvironmental Factors

While most existing research on risk factors for suicide in adolescents focuses on traditional cognitive factors (e.g., hopelessness and depression; Gallagher & Miller, 2018), very few studies have examined how socioenvironmental factors associate with suicide ideation in adolescents. Data are also lacking on these factors for adolescents in rural areas where resources are limited. Existing research highlights lack of social support and lack of access to screening, diagnosis and treatment for mental health in rural areas (Runkle et al., 2023). Therefore, it is imperative to also examine these factors which occur in the broader environment and are not necessarily under the control of the adolescent. The IMV model proposes that environment and life events are distal factors that increase vulnerability to suicide ideation; however, specific factors are not explicated in the IMV model. The current study identifies important socioenvironmental factors such as socio-economic status (SES), food and housing security, and access to health care. All can be conceptualized as economic supports, identified by the CDC as aspects of stability that decrease stress, financial strain, and suicide risk (Stone et al., 2017). Youth with stable housing report lower suicide risk than youth who experience homelessness with their families (Barnes et al., 2018). Similarly, child hunger is a robust predictor of suicide ideation in late adolescence and early adulthood (McIntyre et al., 2013); conversely food security was associated with decreased risk for suicide behaviors in adolescents regardless of ethnicity or sex (Brown et al., 2022). Access to health care is also associated with decreased suicide risk (RAND, 2018; Stone et al., 2017), but availability and accessibility vary in rural and under resourced areas (Michael & Jameson, 2017). Furthermore, rural and small/medium metro areas have lower SES than large metro areas and have experienced a 23–51% increase in poverty since 2000 (Parker et al., 2018), both of which are associated with suicide risk in epidemiological studies (Hoffman et al., 2020).

Regarding SES, adolescents may be unaware of their families’ SES or unable to accurately report objective SES. Considering this, the inclusion of subjective SES, measured by an individual’s subjective view of their status in society, may provide valuable insight. However, few studies have examined the relationship between subjective SES as a measure of social status and mental health-related outcomes. One study utilized the MacArthur Scale of Subjective Social Status (Adler et al., 2000) and found no direct or indirect relationships between adult’s reported subjective SES and depression and anxiety (Galvan et al., 2023). Furthermore, few studies have examined this relationship in adolescents, and the existing findings contrast with those observed in adult samples. For instance, one study employing the youth version of MacArthur scale (Goodman et al., 2001) in a sample of Canadian adolescents found that higher subjective SES was associated with lower odds of suicidal ideation (Sampasa-Kanyinga & Hamilton, 2016). These findings highlight the importance of assessing adolescents perceived social status as a measure of family SES. Additional research is needed on how perceived SES may associate with suicide ideation in adolescents, particularly those in rural, under resourced areas.

The IMV model also specifically identifies lethal means access as a proximal, environmental factor related to suicide ideation persistence and risk for suicide behavior. Having access to lethal means, especially firearms (the leading and most lethal method for suicide attempts in the United States), is a robust proximal risk factor for suicide behavior (Kivisto et al., 2021; Swanson et al., 2021). Specifically, adolescents who live in states with high firearm ownership have significantly higher all-cause (i.e., not limited to a specific method) suicide when compared to peers who live in states with lower firearm ownership (Kivisto et al., 2021). This disparity is likely driven by the ease of access to firearms. Suicide risk is three times higher for individuals living in homes with firearms compared to those without (Anglemyer et al., 2014). Among adolescents, the risk is over four times higher (Swanson et al., 2021). It has been reported that 75% of adolescent suicides occur in the home; of those involving firearms, 79% of the firearms belonged to a family member and 19% belonged to the adolescent (Barber et al., 2022). Recent research from a nationally representative online survey suggests that one-third of adolescents reported being able to access a loaded firearm within five minutes and an additional 17% reported they could gain access in under an hour (Salhi et al., 2021). Access is particularly prevalent in rural areas, where adolescents are more likely to live in households with firearms (Spark et al., 2021), and gain access at a younger age (Caves Sivaraman et al., 2023). Adolescents in rural regions also face higher rates of hospitalization from a self-inflicted injury by firearm (McLoughlin et al., 2019). Given the accessibility and lethality of firearms, further research is needed to examine the role of firearm access and adolescent suicide risk in rural communities.

Social support, especially *perceived* support from family, friends, and school, is another socioenvironmental factor associated with suicide risk (Brausch & Decker, 2014; Victor et al., 2019). Most existing research on social support in the context of adolescent suicide risk focuses on family and peer support, but school and teacher support are also important components (Miller et al., 2015). Limited research shows stronger associations with suicide ideation for parent and school support than for peer support in adolescent inpatients (Miller et al., 2015). Greater perceived social support from peers and parents are associated with lower suicide ideation in many samples of community adolescents (Kalgren & Brausch, 2025; Madjar et al., 2018). However, research on teacher support is more mixed. Some studies find that greater teacher support is associated with lower suicide ideation, while some studies report no association at all (Fredrick et al., 2018; Madjar et al., 2018). School support is often operationalized as general school climate (Stadler et al., 2010) or student involvement/engagement (Young et al., 2011). However, it has yet to be examined in terms of emotional and practical support; for instance, whether school staff or classmates help students solve their problems or ensure they have the things they need. The current study assesses multiple components of social support including perceived support from parents, teachers, friends, classmates, and others in the school to obtain a more holistic picture of social support.

## Current Study

Although prior research has focused on suicide risk factors such as hopelessness and depression, these factors have shown limited utility in predicting future suicidal thoughts and behaviors. Moreover, much of the literature emphasizes cognitive factors while overlooking socioenvironmental factors that may be critical for suicide prevention efforts. Addressing this gap, the current study examined a broad range of cognitive and socioenvironmental factors that may contribute to suicide ideation in adolescents. For cognitive factors, it was hypothesized that defeat and entrapment would positively associate with current suicide ideation, and grit and self-efficacy would negatively associate with suicide ideation. For socioenvironmental factors, it was hypothesized that access to firearms, food and housing insecurity, and lack of access to health care would be positively associated with suicide ideation, and that social support and SES would negatively associate with suicide ideation. In addition to examining and comparing bi-variate correlations, it was also hypothesized that in a multivariate model, grit and self-efficacy would significantly associate with suicide ideation above and beyond defeat and entrapment. Lastly, the socioenvironmental factors were hypothesized to associate with suicide ideation after accounting for the cognitive factors (defeat, entrapment, grit, self-efficacy).

## Method

### Participants

Data were collected from 458 students from two public high schools in the South-Central region of the United States. Both schools are in regions classified as low-income and health care professional shortage areas; one of the schools is in a region classified as rural. The mean age of the sample was 15.64 (*SD* = 1.23). The majority (72.4%) of the sample identified as White, 11.6% identified as Asian, 7.8% identified as Multi-ethnic, 3.6% identified as other, 3.6% identified as Black, 0.9% identified as Native Hawaiian/Pacific Islander, and 0.2% identified as Native American. About half (50.8%) of the sample identified as female, 45.1% identified as male, 1.1% identified as transgender, 1.1% identified as non-binary/gender fluid, 0.9% declined to state their gender, 0.7% responded “not sure,” and 0.4% identified as other. The majority (81.5%) of the sample identified as heterosexual, 5.9% identified as bisexual, 4.5% responded “not sure,” 3.4% declined to state their sexual orientation, 3.1% identified as gay/lesbian/homosexual, and 1.7% identified as other.

### Procedure

The study was approved by the Institutional Review Board at Western Kentucky University. Recruitment of adolescents used the following criteria. *Inclusion criteria*: (1) being a student enrolled in 9th -12th grades at schools from which participants were recruited, (2) being between the ages of 14–18, and (3) having the ability to read and speak English. *Exclusion criteria*: factors that would impair an individual’s ability to provide assent and participate fully in the study, such as intellectual disability, active psychosis, or intoxication.

Different recruitment methods were used for the two schools due to their preference for distributing consent forms to parents. At one high school, 1400 parent consent forms were sent home with all students at the beginning of the year to be signed and returned. Of these, 874 were returned (~ 62% return rate). Of those returned, 527 were positive (60%), 272 were negative 31%), and 75 (9%) signed the form but did not indicate positive/negative consent and did not respond to further communication. At the other high school, a link to an electronic consent form (on Qualtrics) was emailed to parents of all students (*n* = 900). Of these, 87 were completed (~ 10% return rate). Of those returned, 78 were positive (91%), eight (9%) were negative, and one parent signed but did not indicate the students’ name (< 1%). Once informed consents from parents were collected, data collection days were arranged with the schools. On each day, adolescents were called down from their classes, received information about the study, and could choose to participate in the study or decline. Of the 605 positive parent consent forms returned, 456 of those adolescents were present on data collection days and assented to participate. Of those who did not participate, 48% declined, 22% were no longer enrolled at that school, and 22% were absent. Data from three participants were excluded from the study after indicators of potential intellectual disability, based on consultation with school counselors familiar with these students. Additionally, data from five participants were excluded from the study after determining their English language proficiency was insufficient, also determined in consultation with school counselors.

School counselors secured space at the schools for multiple data collection days at each school. The research team (post-bac technician, graduate, and undergraduate research assistants) visited the schools and administered the research protocols. Participants were provided with information about the study and assent forms. In small groups (6–8), participants completed the protocol which took around 30–45 min to complete. Data collection occurred in available rooms at the school where participants were spaced apart from one another to ensure privacy. The research team remained in the room while participants completed protocols to answer questions and provide any additional instructions. After completing the research protocol, participants received monetary compensation ($5) for their participation, a debriefing sheet including local crisis resources, and guidance counselors’ contact information and returned to class.

The research team screened all participant data before leaving the school each day to identify those who endorsed pre-identified critical items for suicide risk. For those who endorsed critical items, the team completed a referral form that briefly described the nature of the risk (e.g., report of recent suicide attempt and/or report of recent, serious thoughts about suicide). Participants who endorsed critical items were confidentially referred to school and/or crisis counselors at their school for further assessment and referral needs on the same day. Participating schools implemented their existing protocols for managing students in crisis. A total of 70 (15.4%) participants were referred; 32.9% were high risk (reported recent frequent thoughts of suicide and/or made a suicide attempt in the past 1–6 months), 35.7% were moderate risk (reported thoughts of suicide in past monthand/or made a suicide attempt in the past 6–12 months), and 31.4% were low risk (reported thoughts of suicide in past 6 months, no history of attempts).

### Measures

#### Suicidal Ideation Severity

Suicidal ideation severity was assessed with the Suicide Ideation Questionnaire- Junior (SIQ-JR; Reynolds, 1987). The SIQ-JR is a 15-item self-report measure of an adolescent’s suicide ideation in the past month, designed for use with adolescents in grades 7–12. Items are rated according to a 7-point scale ranging from 6 (almost every day) to 0 (I never had this thought). A sample item is “I thought it would be better if I was not alive.” Total scores range from 0 to 90 with higher scores indicating a greater intensity of suicide ideation. In the current sample, 3.9% of participants scored at or above the clinical cutoff score of 31. The SIQ-JR has demonstrated good internal consistency (α = 0.94 to 0.97), and adequate concurrent and construct validity (Pinto et al., 1997). In the present study, the internal consistency reliability calculated with Cronbach’s alpha was 0.94.

#### Self-Efficacy

Self-efficacy was assessed with the General Self-Efficacy Scale (GSE; Schwarzer & Jerusalem, 1995). The GSE is a brief, 10-item measure that assesses general self-efficacy and has shown good internal consistency (α = 0.76–0.90). Items are rated on a 4-point scale ranging from 1 (Not true at all) to 4 (Exactly true). A sample item is “I can always manage to solve difficult problems if I try hard enough.” Total scores range from 10 to 40, with higher scores indicating more self-efficacy. The GSE has been used in multiple countries with both adolescents and adults (Luszczynska et al., 2005). In the present study, the internal consistency reliability calculated with Cronbach’s alpha was 0.88.

#### Grit

Grit was assessed with the Short Grit Scale (Grit-S; Duckworth & Quinn, 2009). The Grit-S is an 8-item measure that assesses perseverance and passion for long-term goals. Items are rated on a 5-point scale ranging from 1 (Very much like me) to 5 (Not like me at all). Sample items include: “I am diligent” and “I finish whatever I begin.” Total scores range from 8 to 40, with higher scores indicating greater grit. The Grit-S was found to have good test-retest reliability (*r* = .68) and internal consistency (*r* = .82-0.84) in a diverse sample of adolescents. Cronbach’s alpha for the current study was 0.75.

#### Entrapment

Entrapment was assessed with The Entrapment Scale (Gilbert & Allan, 1998) which contains 16 items and measures both internal (“I want to get away from myself”) and external (“I am in a situation I feel trapped in”) entrapment. Items are rated on a 5-point scale ranging from 0 (Not like me at all) to 4 (Extremely like me). Total scores range from 0 to 64, with higher scores indicating a stronger sense of entrapment. The scale has high internal consistency for young adults (α = 0.88-0.93) and has been validated for use with adolescents (α = 0.91-0.94; Russell et al., 2018). In the current study, the internal consistency reliability was 0.94.

#### Defeat

Defeat was assessed with The Defeat Scale (Gilbert & Allan, 1998) which contains 16 items assessing feelings of defeat, described as a sense of “failed struggle and losing rank.” Items are rated on a 5-point scale ranging from 0 (Not like me at all) to 4 (Extremely like me). Total scores range from 0 to 64, with higher scores indicating a stronger sense of defeat. One sample item includes: “I feel that there is no fight left in me.” The measure was successfully validated in an adolescent sample (α = 0.95; Russel et al., 2018). Cronbach’s alpha in the present sample was 0.94.

#### Perceived Social Support

Perceived social support was assessed with the Child and Adolescent Social Support Scale (CASSS; Malecki & Demaray, 2002). The CASSS includes 60 items across five subscales that assess perceived support from parents, teachers, classmates, close friends, and their people in their school. The CASSS was developed and has been validated for children and adolescents in grades 3–12. Items are rated on a 6-point scale ranging from 1 (Never) to 6 (Always). One sample item is “My parents show they are proud of me.” In diverse samples of high school students, internal consistencies are excellent (α = 0.96-0.97 for all subscales; Malecki & Demaray, 2014). Reliability for the present sample for the CASSS total score was 0.98. Cronbach alphas for the CASSS subscales were also excellent, ranging from 0.94 to 0.97.

#### Food and Housing Insecurity/Access To Health Care

Items from the Youth Risk Behavior Surveillance System (CDC, 2025b) were used to assess food and housing insecurity, as well as access to health care. The item assessing food insecurity asked, “*During the past 30 days*,* how often did you go hungry because there was not enough food in your home?*” Responses include 1 (Never), 2 (Rarely), 3 (Sometimes), 4 (Most of the Time) and 5 (Always). This item was treated as a continuous variable in analyses, with a higher score indicating greater food insecurity. The item assessing housing stability asked, “*During the past 30 days*,* where did you usually sleep?*” Responses include options such as with parents in our home, home of friend/family members, shelter, motel/hotel, and car/park. Almost all participants (99%) reported staying in their parent’s/guardian’s residence. This item was not included in analyses due to very low variability in responses. Two items were used to assess healthcare access. One item asked, “*When was the last time you saw a dentist for a check-up*,* exam*,* teeth cleaning*,* or other dental work?*” The second item asked, “*When was the last time you saw a medical doctor (pediatrician*,* family medicine doctor*,* etc.) for a check-up*,* injury*,* illness*,* or other medical reason?*” Both items had the following response options: “During the past 12 months,” “Between 12 and 24 months ago,” “More than 24 months ago,” “Never,” and “Not Sure.” These items were summed to create a “medical lapse” variable, with higher scores indicating greater lapses in medical care. Lastly, one item assessed current health insurance status: “*Does your family currently have health insurance from a job or through Medicaid/Medicare?*” The response options for this item included “Yes,” “No,” and “Not Sure.” Most (81.6%) reported that they did have insurance, 3.3% said they did not, and 15.1% said they were not sure. This item was also not included in analyses due to the large number of “not sure” responses, and limited variability in those who did and did not currently have insurance.

#### Socioeconomic Status (SES)

Socioeconomic status (SES) was assessed using both an objective measure, an item assessing annual family income, and a subjective measure of perceived social status using the MacArthur Scale of Subjective Social Status–Youth Version (SSS; Goodman et al., 2001). The item measuring objective SES asks participants “Giving your best estimate, what is your annual family income (each year)?” Responses to this question range from $20,000 or less to $150,000 or more. However, 28% of participants selected the response “I prefer not to answer this question.” The SSS scale depicts a ladder with 10 rungs with the following directions: “*Imagine that this ladder pictures how American society is set up. At the top of the ladder are the people who are the best off - they have the most money*,* the highest amount of schooling*,* and the jobs that bring the most respect. At the bottom are people who are the worst off - they have the least money*,* little or no education*,* no job*,* or jobs that no one wants or respects. Now think about your family. Please tell us where you think your family would be on this ladder. Mark the rung that best represents where your family would be on this ladder*.” Adolescents mark one rung of the ladder, which are coded from 1 (bottom rung) to 10 (top rung). Adolescents’ subjective SES ratings may be more accurate than their knowledge of objective SES. This measure has been validated in several diverse samples of adolescents (Quon & McGrath, 2014). Since close to one-third of participants did not provide a family income for objective SES, only the subjective SES item was used in analyses.

#### Access To Firearms

To assess access to firearms, one question was asked, “*How many guns that are in working condition do you have in your house*,* including handguns*,* rifles*,* and shotguns?”* Six response options were provided ranging from “None” to “Five or more.” Access to firearms was coded as no (0) for responses of none, and yes (1) for responses of one or more.

### Data Analytic Plan

To address hypothesis 1, we examined relations between the eight potential correlates of suicidal ideation. Consistent with prior work (Brausch et al., 2025a, b), SIQ total scores were transformed to reduce skewness and kurtosis (i.e., arcsine(sqrt(SIQ_Total + 1)/100); unadjusted skewness and kurtosis = 2.72 and 9.55, respectively; transformed skewness and kurtosis = 1.52 and 2.84, respectively). Adjusting these relations by sex were originally considered; however, analyses indicated trivial differences in the estimates of the *r*s and standard errors when comparing the zero-order correlations to standardized estimates from GLMs that included sex as a covariate (all absolute difference in *r*s < = 0.013 with identical patterns of statistical significance). Given these trivial differences, zero-order correlations between suicidal ideation and the potential correlates are presented for simplicity. To estimate missing data, multiple imputation was used in Mplus (n datasets = 10), though all estimates were very similar, and all inferential conclusions were identical when compared to using listwise deletion.

Following these analyses, a series of multivariate hierarchical regressions were conducted to determine contributions to unique variance in suicidal ideation. At the first step, defeat and entrapment were entered. At the second step, grit and self-efficacy were included as covariates. Finally, the remaining socioenvironmental variables (e.g., social support) were included.

## Results

As shown in Table 1, all variables, except for access to firearms, significantly correlated with suicidal ideation. Among all the significant correlations, effect sizes ranged from (in absolute terms) from 0.17 (lapse in medical support) to entrapment (0.68). No adjustments for multiple testing were conducted since all significant *p* values were below 0.0001 (and thus would be robust to conservative adjustments for Type 1 error). Follow-up tests comparing the high effect size correlations of entrapment (0.68) and defeat (0.65) were significantly higher than all other correlations shown in Table X (all *p*s < 0.001).

**Table 1:** Zero-order correlations between suicidal ideation and potential correlates

	Suicidal Ideation		
	*r*	S.E.	*p*-value
Defeat	0.65	0.028	< 0.0001
Entrapment	0.68	0.026	< 0.0001
Grit	-0.35	0.042	< 0.0001
Self-Efficacy	-0.35	0.041	< 0.0001
SES	-0.24	0.046	< 0.0001
Firearms	0.03	0.047	0.56
Social Support	-0.45	0.038	< 0.0001
Food Insecurity	0.25	0.045	< 0.0001
Medical Lapse	0.17	0.048	< 0.0001

Multiple imputation *n* = 457

The multivariate hierarchical models indicated that both defeat (β = 0.29, *p* < .001) and entrapment (β = 0.45, *p* < .001) were significant predictors of suicidal ideation when examined simultaneously, accounting for 48.3% of the variance in suicidal ideation. Including grit (β = − 0.03, *p* = .49) and self-efficacy (β = − 0.03, *p* = .53) resulted in virtually the same r-square as the prior model (48.4%), suggesting the variables do not contribute to the prediction of suicidal ideation above and beyond defeat and entrapment (whose estimates remained virtually unchanged; βs = 0.27 and 0.44, respectively). At step 3, only defeat (β = 0.25, *p* < .001), entrapment (β = 0.41, *p* < .001), and food insecurity (β = 0.12, *p* < .01) were statistically significant; the model r-square of 50.2% was only slightly higher than the model that only included defeat and entrapment and virtually identical to a follow-up model that only included defeat, entrapment, and food insecurity (r-square = 49.5%; see Table 2). In sum, there was little evidence that the other variables provided meaningful unique variance accounted for above defeat and entrapment, with only food insecurity maintaining its statistical significance within the multivariate models.

**Table 2:** Hierarchical regression predicting suicidal ideation

Predictor	β	*p*-value	Model *R*^2^	ΔR²	
Step 1					
Defeat	0.29	< 0.001			
Entrapment	0.45	< 0.001	0.483		
Step 2					
Defeat	0.27	< 0.001			
Entrapment	0.44	< 0.001			
Grit	-0.03	0.49			
Self-Efficacy	-0.03	0.53	0.484	0.001	
Step 3					
Defeat	0.25	< 0.001			
Entrapment	0.41	< 0.001			
Grit	-0.03	0.52			
Self-Efficacy	0.001	0.97			
SES	-0.02	0.62			
Firearms	0.05	0.15			
Social Support	-0.06	0.19			
Food Insecurity	0.12	<0.01	0.502	0.018	

Multiple imputation *n* = 441

## Discussion

Decades of research on suicide risk in adolescents have primarily focused on traditional risk factors such as depression, hopelessness, and psychiatric diagnoses. However, these traditional factors have limited effectiveness at predicting suicidal thoughts and behaviors (Fox et al., 2015; Franklin et al., 2017). Existing literature also focuses on cognitive factors while underscoring the potential importance of socioenvironmental factors, which has been emphasized as an area in need of further research (e.g., Glenn et al., 2017; Gallagher & Miller, 2018). To address this gap in the literature, the current study examined a broad range of cognitive (i.e., defeat, entrapment, grit, self-efficacy) and socioenvironmental factors (i.e., SES, access to firearms, food insecurity, lapses in medical care, social support) that may contribute to suicide ideation in adolescents. Our study also owes much to the IMV model (O’Connor & Kirtley, 2018) that emphasizes the dynamic and complex pathway through which risk for suicide ideation and attempts may be conferred. We hypothesized that cognitive factors, such as defeat and entrapment, would positively associate with current suicide ideation, and grit and self-efficacy would negatively associate with suicide ideation. We also hypothesized that socioenvironmental factors, such as access to firearms, food and housing insecurity, and lack of access to health care would be positively associated with suicide ideation, and that social support and SES would negatively associate with suicide ideation. Lastly, we hypothesized that all factors would significantly associate with suicide ideation after accounting for defeat and entrapment.

First, we found that both defeat and entrapment were significantly and positively correlated with suicidal ideation severity. Notably, these variables demonstrated the strongest associations with suicide ideation compared to all other variables. In the multivariate model, defeat and entrapment were significantly associated with suicide ideation even after all other cognitive and socioenvironmental factors were accounted for, indicating their prominence as risk factors for thoughts of suicide. Although few studies have examined defeat and entrapment in adolescent samples, our findings align with those that do exist (Pollak et al., 2021; Yang et al., 2022). Specifically, Park and colleagues (2010) also found entrapment to be most strongly associated with suicidal ideation compared to trait anger, psychosomatic symptoms, and depression in a Korean adolescent sample; however, defeat was not measured. Defeat and entrapment have been examined among adult samples, showing robust relationships between defeat and entrapment and suicidal ideation above and beyond other risk factors (Siddaway et al., 2015; Silvestre Vidal et al., 2024). Defeat and entrapment are central components of the IMV model, indicating that some people experience the emergence of suicidal ideation to process feelings of defeat and entrapment, and some may even experience tunnel vision, where suicide seems like the only way out. This process may be particularly relevant during adolescence where defeat may stem from social rejection (O’Connor & Kirtley, 2018). Entrapment is also a well-known risk factor for suicide, in which individuals believe there is no way out of their situation or terrible state of mind (Park et al., 2010). Results from the current study add to the existing literature by demonstrating similar associations between both defeat and entrapment and suicide ideation in adolescents, highlighting that these relationships are not unique to adults. These findings also provide support for the IMV model of suicidal behavior, identifying the critical role that defeat and entrapment play in the emergence and maintenance of suicide ideation in youth. Current study results provide evidence for what may be a universal experience in terms of feelings of defeat and entrapment relating to suicide ideation, highlighting what may be a prominent emotional and cognitive experience that contributes to development of suicide ideation in adolescents.

Grit and self-efficacy were additional cognitive factors examined in this study and are noted as more novel potential protective factors within existing literature. Current study results found that both grit and self-efficacy were significantly and negatively correlated with suicidal ideation severity. While grit has cross-sectionally and longitudinally associated with suicidal ideation among adults (Clement et al., 2020; Manuel, 2023), it was less clear if and how grit would associate with suicide ideation in adolescents. Existing research on grit in adolescent samples has shown positive associations with academic achievement and meaning in life (Clark et al., 2020; Datu et al., 2016); however, few studies have directly examined the association between grit and suicidal ideation in this population. Our results expand upon the limited prior research on grit and suicide ideation in Chinese adolescents with an absent parent, showing that higher levels of grit associate with less severe suicide ideation. A major component of grit is identifying and pursuing long-term goals; therefore, we expected a similar relationship between suicide ideation and self-efficacy, an adolescent’s belief in their ability to achieve their goals. Current study findings align with previous research, showing a negative association between self-efficacy and suicidal ideation (Li & Kwok, 2023; Wu & Yaacob, 2017). However, neither grit nor self-efficacy was associated with suicide ideation when defeat and entrapment had been accounted for in multivariate analyses. It may be that these qualities have indirect relationships with suicide ideation and might serve as protective factors in the relationship between defeat and entrapment and suicide ideation. For example, higher self-efficacy is indicative of problem-solving skills and less hopelessness, which are also known to buffer from suicide risk (Gómez-Tabares et al., 2022; Ribeiro et al., 2018). Examinations of grit and self-efficacy as moderators and longitudinal data are needed to evaluate the protective and directional role of these cognitive factors.

This study also examined the relationship between socioenvironmental factors and suicidal ideation severity. While all were significantly correlated with suicide ideation (except firearm access), they were not as strongly associated with suicide ideation as defeat and entrapment. Furthermore, only food insecurity was associated with suicide ideation once all other factors were accounted for. These findings are consistent with previous research linking food insecurity to increased risk of suicidal thoughts and behaviors in adolescents (e.g., Brown et al., 2022). Results from the current study extend this literature by demonstrating this association in a rural, underserved sample, where food insecurity may co-occur with other resource limitations that elevate suicide risk. The fact that food insecurity emerged as the only significant socioenvironmental factor to associate with suicide ideation above and beyond defeat and entrapment indicates that lack of sufficient food and experiencing hunger may represent a tangible, immediate, and consistent reminder of a disadvantaged economic situation. It may be that adolescents are more aware of food insecurity on a regular basis, which is also likely related to other economic hardships that may not be as prominent in adolescent awareness such as lower SES and limited access to health insurance and health care. Future research can continue to examine how socioenvironmental factors relate to one another and to suicide risk to improve our understanding of their impact.

Similar to grit and self-efficacy, all other socioenvironmental factors were no longer significantly associated with suicide ideation after accounting for defeat and entrapment in multivariate analyses. However, given the limited existing research on how socioenvironmental factors relate to suicide ideation in rural adolescents, it still seems important to note which factors showed significant bi-variate correlations with suicide ideation. For example, associations between longer intervals since doctor’s and dentist’s visits and greater suicidal ideation severity are consistent with research indicating that limited access to healthcare services may increase vulnerability to suicidality (e.g., Tondo et al., 2006). For medical care, longer intervals since a doctor’s visit may mean missed opportunities for mental health screening and early intervention, both of which are critical for preventing suicidal behaviors (Hua et al., 2023). However, adolescents with a history of suicidal ideation often underutilize mental health services (LeCloux et al., 2017), and youth in rural areas face additional barriers such as limited screening for mental health challenges and provider shortages (Fiske et al., 2005; Runkle et al., 2023). These factors may operate in a feedback loop, where suicidal ideation both contributes to and is worsened by reduced engagement with care. However, this association was not strong enough in the current study to associate with suicide ideation above and beyond the experiences of defeat and entrapment. It may be that socioenvironmental factors are associated with defeat and entrapment, which are then associated with suicide ideation. Future research can examine these potential indirect pathways more specifically.

In contrast, firearm access was not significantly associated with suicidal ideation severity in bivariate or multivariate analyses. While prior research has consistently linked the presence of firearms to higher rates of adolescent suicide deaths (e.g., Kivisto et al., 2021; Swanson et al., 2021), fewer studies have focused specifically on the relationship between firearm access and suicidal thoughts in adolescents (Kemal et al., 2023). The current findings align with a study by Simonetti and colleagues (2015), which found that suicidal thoughts did not differ between adolescents with and without firearm access. Taken together, these findings suggest that firearm access may not be a critical factor during the ideation stage but could become more influential when adolescents begin forming a plan or take steps toward an attempt. For adolescents experiencing ideation without a plan or intent, access to firearms may not significantly influence the severity of their thoughts. The finding from the present study contributes to the literature by specifically examining how firearm access relates to suicidal ideation severity in adolescents in rural areas, where exposure to guns often begins at a younger age (Caves Sivaraman et al., 2023). As the most common method for suicide death in adolescents is firearms (Kim et al., 2025), future research should investigate whether firearm access plays a more significant role in the transition from ideation to planning or attempts. Understanding the stage-specific influence of firearm access may be key to developing more targeted prevention strategies in rural and underserved adolescent populations.

Lastly, total perceived social support was significantly and negatively correlated with suicidal ideation severity, aligning with previous research demonstrating the protective role of social support in suicidal ideation for adolescents (Brausch & Decker, 2014; Victor et al., 2019). However, like most other factors examined in the current study, social support was not associated with suicide ideation after accounting for defeat and entrapment. In the IMV model, social support is proposed as a motivational moderator in the relationship between entrapment and suicide ideation. Interestingly, according to a recent systematic review of existing research evaluating the IMV model, there were no identified studies that assessed social support within the central pathway of the model (Souza et al., 2024). The closest variable to social support that had been studied was thwarted belongingness, which can be defined as loneliness, isolation, and not feeling socially integrated. A few studies examined thwarted belongingness in the relationship between entrapment and suicide ideation, but it was not a significant moderator (Forkmann & Teismann, 2017; Wetherall et al., 2022). The review by Souza and colleagues also summarized cross-sectional studies that evaluated IMV constructs, and a few were included that examined differences in social support between individuals with no suicide ideation or attempt history, those with ideation history, and those with attempt history. All of the studies found social support to be higher among individuals with no suicide history across various samples of community adults (Branley-Bell et al., 2019; Seidler et al., 2023; Wetherall et al., 2018) and adolescents (del Carpio et al., 2020; Nestor et al., 2022). Results from the current study seem to be in line with the limited existing research on social support within the IMV model, in that while bivariate correlations are found, social support did not meaningfully add to the model explaining suicide ideation after accounting for defeat and entrapment. Given the limited existing literature on social support within the IMV model, further research is needed to better understand how it may or may not provide a buffer between entrapment and suicide ideation. Future research can also examine how perceived social support from different sources (e.g., close friends, parents, teachers) may impact these relationships since it has been noted that support from parents and classmates is more protective for suicide than other sources of support (Kalgren & Brausch, 2025).

### Limitations

Although this study expands our understanding of cognitive and socioenvironmental factors that associate with suicidal ideation amongst adolescents in rural, under-resourced communities, the current study does have limitations. First, data were collected cross-sectionally and determining directionality is not feasible. While we did aim to assess suicide ideation and other factors within similar, recent timeframes, future research should examine these factors longitudinally to identify potential risk and/or protective factors. Additionally, the sample lacks racial and ethnic diversity which limits generalizability of our results. Racially or ethnically minoritized adolescents may experience higher levels of adversity and socioeconomic disparities leading to increased challenges with suicidal ideation. This limitation, however, does reflect the geographic area in which data were collected and the shortage of health professionals in the community. Lastly, self-report data collection methods were used exclusively for the study. While self-report data is valuable to examine the influence of variables such as perceived SES, participants may misreport or consciously skew answers for desirability. As a result of the sensitive question material, self-report bias can be minimized by ensuring anonymity and confidentiality to participants.

## Conclusion

Existing literature on risk and protective factors for suicide ideation in adolescents focuses on homogenous risk factors that have not advanced understanding of adolescent suicide risk. Addressing this gap, the current study evaluated how cognitive factors such as defeat, entrapment, self-efficacy, and grit would either positively or negatively associate with suicide ideation. The present study expanded the adolescent suicide literature by demonstrating the central role of defeat and entrapment, alongside food insecurity, in shaping suicide ideation severity among youth in rural and under-resourced communities. While multiple cognitive and socioenvironmental factors were associated with suicide ideation at the bivariate level, defeat and entrapment emerged as the most robust predictors in multivariate analyses, underscoring their salience above other commonly cited risk and protective factors. The unique contribution of food insecurity further highlights the importance of addressing basic unmet needs when assessing suicide risk in rural adolescents. Together, these findings suggest that prevention and intervention efforts may benefit from prioritizing cognitive experiences of entrapment and defeat while also incorporating strategies to reduce material hardship. Future research should continue to explore how cognitive and socioenvironmental factors interact over time to inform more targeted, contextually responsive suicide prevention approaches for rural youth.

## Data Availability

Data used in this study are available through the NIMH Data Archive (collection #C4919).
